# Dendritic Spines Shape Analysis—Classification or Clusterization? Perspective

**DOI:** 10.3389/fnsyn.2020.00031

**Published:** 2020-09-30

**Authors:** Ekaterina Pchitskaya, Ilya Bezprozvanny

**Affiliations:** ^1^Laboratory of Molecular Neurodegeneration, Institute of Biomedical Systems and Biotechnology, Peter the Great St. Petersburg Polytechnic University, St. Petersburg, Russia; ^2^Department of Physiology, UT Southwestern Medical Center at Dallas, Dallas, TX, United States

**Keywords:** dendritic spines, neuronal morphology, mushroom spine, thin spine, stubby spine, classification, clusterization

## Abstract

Dendritic spines are small protrusions from the dendrite membrane, where contact with neighboring axons is formed in order to receive synaptic input. Changes in size, shape, and density of synaptic spines are associated with learning and memory, and observed after drug abuse in a variety of neurodegenerative, neurodevelopmental, and psychiatric disorders. Due to the preeminent importance of synaptic spines, there have been major efforts into developing techniques that enable visualization and analysis of dendritic spines in cultured neurons, in fixed slices and in intact brain tissue. The classification of synaptic spines into predefined morphological groups is a standard approach in neuroscience research, where spines are divided into fixed categories such as thin, mushroom, and stubby subclasses. This study examines accumulated evidence that supports the existence of dendritic spine shapes as a continuum rather than separated classes. Using new approaches and software tools we reflect on complex dendritic spine shapes, positing that understanding of their highly dynamic nature is required to perform analysis of their morphology. The study discusses and compares recently developed algorithms that rely on clusterization rather than classification, therefore enabling new levels of spine shape analysis. We reason that improved methods of analysis may help to investigate a link between dendritic spine shape and its function, facilitating future studies of learning and memory as well as studies of brain disorders.

## Introduction

Dendritic spines are tiny protrusions from dendrites, which form functional contacts with neighboring axons of other neurons (Smith et al., 2014). Dendritic spines are very plastic and their size and shape are constantly changing in response to neuronal activity. Complex machinery composed of various signaling molecules and cascades maintains the unique structure and function of dendritic spines (Yasuda, 2017; Nakahata and Yasuda, 2018). Dendritic spine shape is controlled by the actin cytoskeleton. A characteristic feature of excitatory spines is a postsynaptic density (PSD), which is visible on electron microphotographs. PSD consist of densely packed ion channels, receptors, and kinases/phosphatases anchored by scaffolding proteins. Learning and memory formation processes are tightly linked to remodeling or elimination of existing dendritic spines and formation of new ones, which enables modulation of information transfer efficiency between neurons (Yang et al., 2014; Segal, 2017; Chidambaram et al., 2019; Stein and Zito, 2019). For example, motor learning induces rapid growth of new dendritic spines at mice contralateral motor cortex neurons, and subsequent elimination of spines existing before training, so the overall spine density is relatively constant (Xu et al., 2009). For all these reasons, dendritic spines are believed to serve as sites for memory formation and storage, initiating memory consolidation through mechanisms of potentiation and depression of synaptic activity (Zhou et al., 2004; Bourne and Harris, 2007; Kasai et al., 2010; Bailey et al., 2015).

Changes in dendritic spines were detected after being subject to various stimuli, including drug administration (Barrientos et al., 2018), hypoxia (Saraceno et al., 2012), environmental changes (Ashokan et al., 2018), neurodevelopmental (Nishiyama, 2019), neurodegenerative (Herms and Dorostkar, 2016) and psychiatric diseases (Penzes et al., 2011) and many others. Neurodegenerative disorders are characterized by synapse loss and dendritic spine abnormalities in the brain region associated with the disease. For example, Alzheimer’s disease is known to be accompanied by dendritic spine shrinkage and elimination in the hippocampal and cortexes areas, which is proposed to start before any clinical evidence of the disease, like cognitive decline and memory dysfunction, manifests (Tackenberg et al., 2009; Boros et al., 2017). Genetic neurodegenerative disorder Huntington’s disease is characterized by synapse loss in the striatal brain region, which is linked with progressive movement discoordination (Nithianantharajah and Hannan, 2013). In contrast, autism spectrum disorders are characterized by a significant increase in spine density on various brain areas, including frontal, temporal, and parietal lobes and lateral nucleus of the amygdala (Nishiyama, 2019). Compared with healthy subjects, patients with Fragile X syndrome have elevated numbers of dendritic protrusions in the cingulate, temporal, and visual cortex with prevalence of immature one (Bagni and Zukin, 2019; Nishiyama, 2019). The balance between spine appearance, maturation, elimination, and plasticity is critical for proper brain function. Methods for analyzing and understanding the morphology of dendritic spines are critically important for many fields of neuroscience. In this mini-review article, we discuss recent developments, approaches, and software tools that facilitate analysis of complex dendritic spine morphology on microscopic images obtained from cultured neurons, fixed brain slices, and intact brain tissue.

## Dendritic Spines Shape Classification and Its Limitations

**Figure 1:**
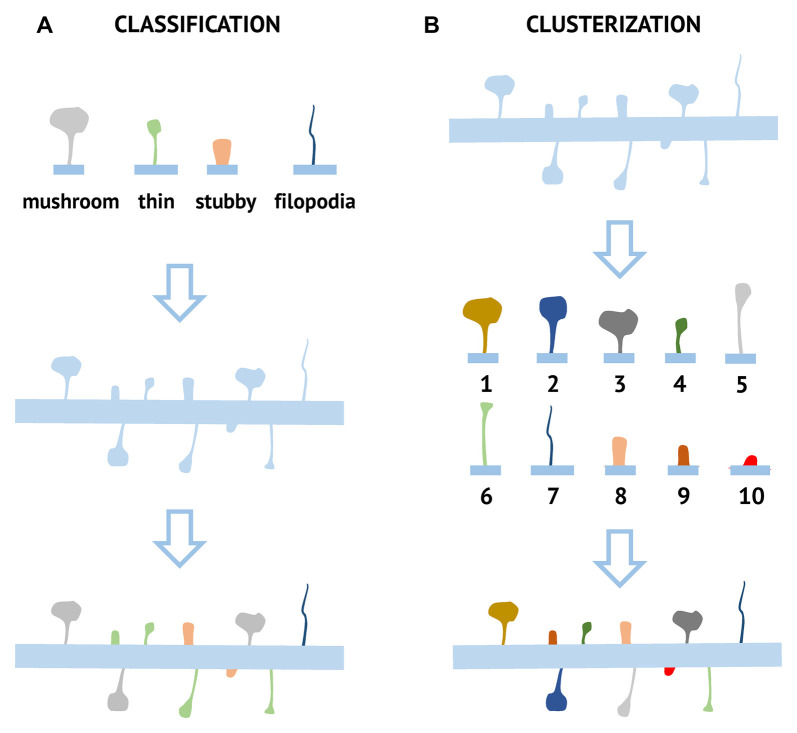
Comparison of classification and clusterization pipelines used for the analysis of dendritic spine morphology. For classification approach **(A)** several possible morphological spine types (mushroom, stubby, thin, and filopodia) are defined based on pre-determined criteria. Each spine is then assigned to one of these classes based on numerical morphological criteria. For the clusterization approach **(B)**, spines are grouped into clusters based on their common morphological features. Ten different clusters (1–10) are shown as an example, but the number of different clusters and parameters used for clusterization depends on the particular algorithm and dataset.

A synapse is a zone of specialized contact between two neurons, serving to transmit information from cell to cell. Most synapses are formed between the axonal bouton and the dendritic spine, which is a specialized protrusion from the dendritic membrane. Dendritic spines come in a variety of shapes and sizes, differing greatly across brain areas, cell types, and animal species (Ghani et al., 2017). During structural analysis dendritic spines are traditionally grouped into four fixed classes according to their morphological features reflecting head and neck properties: mushroom, thin, stubby, and filopodia (Figure 1). Mushroom spines have a large head and a small neck, separating them from a dendrite. They form strong synaptic connections, have the longest lifetime, and therefore are thought to be sites of long-term memory storage (Hayashi and Majewska, 2005; Bourne and Harris, 2007). Thin spines have a structure similar to the mushroom spines, but their head is smaller relative to the neck. They are more dynamic than mushroom spines and believed to be “learning spines,” responsible for forming new memories during the synaptic plasticity process, accompanied by head enlargement (Hayashi and Majewska, 2005; Bourne and Harris, 2007). Stubby spines typically do not have a neck. They are known to be the predominant type in the early stages of postnatal development but are also found in small amounts in adulthood, where they are likely formed due to the disappearance of mushroom spines (Hering and Sheng, 2001). Filopodia are long, thin dendritic membrane protrusions without a clear head, commonly observed in developing neurons. These spines may also be found in mature neurons, but under specific conditions, for example, induction of plasticity after different types of brain injury (Yoshihara et al., 2009). Compared to other types of dendritic spines, filopodia are very mobile and flexible structures with a short lifetime. On electron micrographs, filopodia in most cases do not have PSD and the neighboring axonal terminal contain only a few synaptic vesicles, indicating that they are not likely to form functional synapses. Because of this, filopodia are usually excluded from spine counts during synaptic density calculation (Berry and Nedivi, 2017). There are also additional spine shape classes which have been named by different research groups such as branched and cup-shaped spines (Maiti et al., 2015), but they are not widely used in the field.

Classification of spines into the mushroom, thin, and stubby was initially performed manually, but this is a very labor-intensive process that is prone to subjective errors. Multiple segmentation and classification algorithms were developed to automatize this process, making it faster, easier, and with minimal bias introduced by an experimenter. Classification is performed using a decision tree based on the estimation of several key parameters, such as the size of the spine head, and the ratio of head to the neck, are the most popular approaches, implemented in a variety of software packages available for free and commercially (Koh et al., 2002; Rodriguez et al., 2008; Son et al., 2011; Swanger et al., 2011; Risher et al., 2014; Basu et al., 2016, 2018; Dickstein et al., 2016). Alternative approaches were developed, based on semi-supervised learning (Shi et al., 2014) and classification in the likelihood ratio space using shape and appearance features characterizing dendritic spine morphology (Ghani et al., 2017).

Despite its wide use, the classification approach described above has serious limitations. The transition between mushroom, thin and stubby spine subtypes occurs abruptly in the classification, but in reality, there is a continuum of spines shapes and sizes (Yuste and Bonhoeffer, 2004; Berry and Nedivi, 2017). This statement is supported by research data about spine morphology organization, examining live and fixed brain tissue (Wallace and Bear, 2004; Arellano et al., 2007; Tonnesen et al., 2014; Loewenstein et al., 2015). A study that focused on the morphology of neurons in layers II and III of a mouse visual cortex discovered that a continuous distribution rather than several discrete peaks were observed for each morphological parameter (Arellano et al., 2007). Distribution of dendritic spine length and head diameter in neurons of cortical layer III was also characterized by unimodal distribution (Wallace and Bear, 2004). No evidence of the existence of defined spine types was obtained in another study performed in the neocortex (Loewenstein et al., 2015). A study of the correlation between spine shape and compartmentalization of synapses indicated a great diversity in spine morphology, which was not consistent with standard classification systems (Tonnesen et al., 2014). Advances in live imaging make it possible to analyze the shape of a particular dendritic spine for a prolonged time, which revealed unique plasticity properties. Even stable, persistent spines are changing continuously in their orientation and shape, which also argue the pros existence of the shape continuum (Berry and Nedivi, 2017).

Analysis of spine morphology is also limited by the resolution limit of light microscopy. The size of dendritic spines usually does not exceed 1,000 nm for its largest dimension in the head, while the sizes of other parts are much smaller. For confocal microscopy resolution, the limit is estimated as half of the excitation wavelength, which is approximately 200–300 nm. For two-photon microscopy, the resolution is even lower due to the longer excitation wavelength used in these experiments. Furthermore, resolution along the z-axis is even lower than in the xy-plane in confocal and 2-photon imaging experiments. Low resolution leads to erroneous measurements of spine shapes, which leads to erroneous dendritic spine classification. For example, spine neck width is believed to be the key factor influencing dendritic spine compartmentalization and efficiency of signal transduction (Tonnesen et al., 2014). However, visualization of such small structures can only be done with super-resolution imaging, which provides much more detailed information about spine shapes than standard light microscopic methods. Super-resolution imaging studies suggest that stubby spines are significantly over-represented in literature, which is the consequence of the low resolution of the neck, as it is small (Tonnesen et al., 2014).

Improvement of dendritic spine visualization methods is an important part of the unbiased assessment of their morphology. Due to the lack of clear boundaries between different spine classes, the same brain sample may yield different ratios of mushroom, thin, and stubby spines, depending on the criteria used to separate these classes from each other, meaning analyses are often biased and poorly reproducible. An attempt to fit the continuous distribution of spine shapes and sizes into pre-defined and rigid categories can result in multiple sources and potential errors. For example, thin and mushroom spines are two classical spine subtypes that have a very similar shape and the only critical parameter by which they can be distinguished is the head size. The head size is proportional to the area of PSD, the number of receptors at postsynapse, and synaptic strength (Kharazia and Weinberg, 1999; Takumi et al., 1999; Ganeshina et al., 2004; Arellano et al., 2007). On another hand, the length and width of the spine neck are related to the magnitude of postsynaptic potential (Tonnesen et al., 2014). The morphology of synapses varies depending on the strength of synaptic contact. Changes in synaptic strength during long-term potentiation and long-term depression are associated, respectively, with enlargement or shrinkage of the spine head (Yuste and Bonhoeffer, 2001; Holtmaat and Svoboda, 2009). During this process, it has been suggested that there is an interconversion between the thin and mushroom spine at a given synapse, however, it is not possible to subjectively define this point because of the continuum of spine sizes and shapes.

An additional source of error is related to the structure of the morphological data. Because imaging data are collected across different neurons from primary cultures or animal species of different sexes, it leads to the generation of a multi-level data structure. Recently published research speculates that using conventional statistical methods for working with such types of data, may lead to the generation of erroneous conclusions, and mixed-effects models have been proposed to correct this (Paternoster et al., 2018).

## Non-Classification Approaches to Dendritic Spine Shapes Analysis

The analysis could be more precise and reliable by considering a continuum of spine shapes and forms and there are potential solutions for these classification problems. It has been proposed that reliance on objectively defined morphological parameters, rather than on classification into subjective shape-based groups, may help to solve some of these problems (Mancuso et al., 2013).

One widely used non-classification approach is the direct measurement of key morphological descriptors. A comparison of classification and direct morphometric measurement accuracy during dendritic spine morphology assessment has demonstrated that the second approach is much more sensitive (Ruszczycki et al., 2012). However, the direct morphometric measurement approach does not provide information about spine shape, which is important for biological function. For example, the measurement of spine head size works well for thin and mushroom spines, where it is well correlated with synaptic strength and PSD area (Kharazia and Weinberg, 1999; Takumi et al., 1999; Ganeshina et al., 2004; Arellano et al., 2007), but in filopodia and stubby type spines the head is not clearly defined and therefore not a key parameter in defining its morphology.

**Figure 2:**
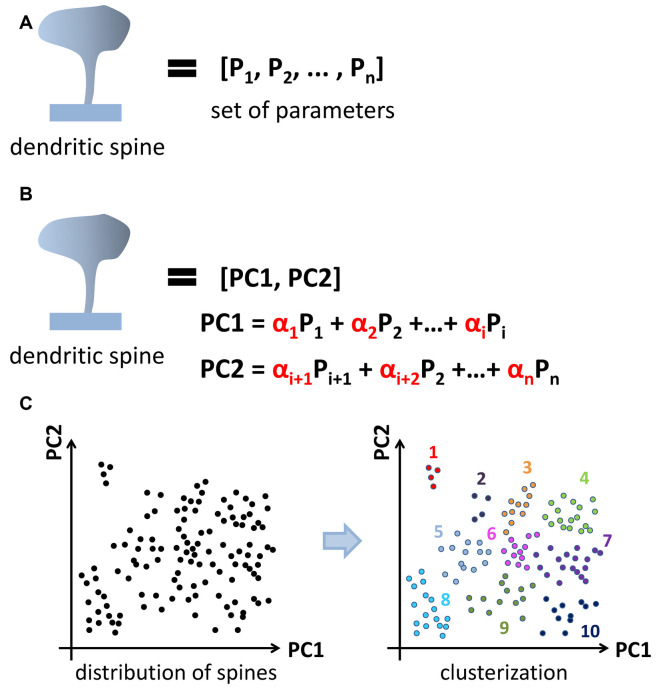
Application of principal component analysis (PCA) method to dendritic spine clusterization. Spine shape is characterized by a set of parameters **(A)**. PCA method is used to reduce data dimensionality before clusterization. Newly generated parameters called principal components composed from initial one and form an orthonormal basis **(B)**. After PCA dataset in new coordinates is subjected to clusterization **(C)**. Cluster shape and content depend on the clusterization method used (k-means, c-means, hierarchical, et cetera).

A newly emerging approach is a clusterization of spines according to similarities in their shape. This “clusterization” approach aims to automatically group spines into similar structural classes based on selected algorithms and without *a priori* input (Figure 1), meaning the results of clusterization are defined by data structure. The spine is presented as a set of values of parameters reflecting its morphology, starting from obvious such as neck and head size to a more complex geometrical parameters that may include a combination of several measurements. The algorithm assesses similarities between spines based on the value of selected parameters and performs clusterization. In this approach principal component analysis (PCA) is used to reduce data dimensionality before clusterization (Figure 2).

**Figure 3:**
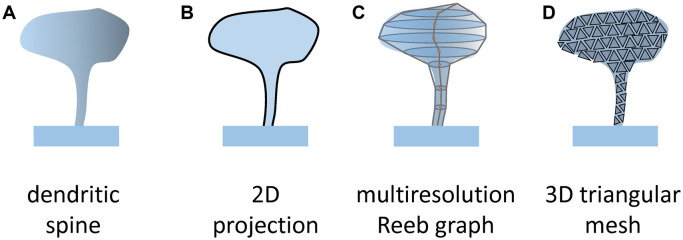
Methods of spines segmentation. To assess numerical values of parameters describing spines shape **(A)** it is necessary to define object boundary. In 2D projection, spines are presented as flat objects (**B**; Ghani et al., 2016; Bokota et al., 2016). In 3D it was proposed to present the spine surface as a set of ellipses along the spine centerline that are connected (**C**; Luengo-Sanchez et al., 2018; Choi et al., 2019) or a triangle mesh (**D**; Kashiwagi et al., 2019). Such a presentation enables us to extract more complex parameters reflecting the constructed model of the spine.

The first practical implementation of the clustering approach was published (Ghani et al., 2016) a few years after its initial suggestion (Mancuso et al., 2013; Table 1). A histogram of oriented gradients (HOG), disjunctive normal shape models (DNSM), morphological features, intensity profile based features, or their combination were used to quantitatively describe dendritic spine shapes. HOG is a feature descriptor used in object recognition and computer vision (Dalal and Triggs, 2005). The input image is divided into small connected parts where gradient direction is counted, visualized as an arrowhead, pointing at discrete angle 0, 30, 60, 90, etc. Later these parts are combined to a larger one and a histogram of obtained gradients is built that describes the analyzed object. DNSM represent a shape as a union of convex polytopes, which are constructed by intersections of half-spaces (Mesadi et al., 2015). Intensity profile based features are built after examination of intensities profile in the regions where the dendritic spine neck is expected to be located (between the head and a dendrite; Erdil et al., 2015). The analysis was performed on 2D maximum intensity projections (Figure 3B) from a 3D stack of two-photon microscopic images due to the non-sufficient resolution along the z-axis. Processing of data was generated with the help of morphological descriptors or their combination by x-means, clustering algorithm results to generate 4 distinct dendritic spines clusters. In all cases at least one of these clusters could not be clearly defined as mushroom, thin, or study spines. Authors concluded that their findings support the idea of existence of intermediate spine shapes. Later, the combination of DNSM and HOG morphological features was used to perform dendritic spine classification with help of a kernel density estimation (KDE) based framework, which enabled analysis of spine shape classes separability in the likelihood ratio space (Ghani et al., 2017).

**Table 1:** Summary of non-classification approaches to dendritic spines shapes analysis.

No	Year	Reference	Sample	Microscopy	Spines form representation (Figure 3)	Preprocessing and feature extraction approach	Number of features	Software (+ provided,− not provided)	Morphological features analysis approach	Software availability
1	2016	Ghani et al. (2017)	7–10 day old mouse brain slices neurons (region not specified)	Two-photon	2D projection from image series	Histogram of oriented gradients (HOG), disjunctive normal shape models (DNSM), morphological features, intensity profile based features or their combination	Varying, from 12 for morphological to 346 for DNSM features	Custom (−)	X-means clustering, number of clusters selected automatically using the Bayesian information criterion (BIC; =4)	NA
2	2016	Bokota et al. (2016)	19–21 days *in vitro* hippocampal neurons	Confocal	2D projection from image series	The most often used morphological features according to literature data	11 (reduced to 6)	Custom (−)	C-means clustering (=10), average-linkage hierarchical clustering (=10), data dimensionality reduction by 2D principal component analysis (PCA)	UR
3	2018	Luengo-Sanchez et al. (2018)	layer III pyramidal neurons of the human cingulate cortex	Confocal	3D triangular surface mesh Multiresolution Reeb graph	Surface of a spine is modeled by 7 segments, which are presented as linked to each other ellipses. From 54 parameters used to describe dendritic spines, 36 reflect ellipses geometry and position and 18 describe more complex features, such as spine growth direction	54	Imaris for segmentation ($), custom software for feature extraction (+)	Clustering by probabilistic model with Gaussian finite mixtures, number of clusters selected automatically using the Bayesian information criterion (BIC; =6)	FA
4	2019	Kashiwagi et al. (2019)	18–22 days *in vitro* hippocampal neurons	SIM	3D triangular surface mesh	Segmentation of spines by multilevel thresholding based on Otsu’s method following geodesic active contour, combination of morphological features and high geometric features	10 (reduced to 5)	Custom (+)	Division into mushroom and non-mushroom spines using SVM classifier, mapping the trajectories of individual spines shape transitions in the feature space, data dimensionality reduction by 3D principal component analysis (PCA)	FA
5	2019	Choi et al. (2019)	18–22 days *in vitro* hippocampal neurons	SIM	3D triangular surface mesh	Processing as in No4 with addition of 5 more features reflecting spines head and neck size	10	DXplorer (−)	K-means clustering, coordinate plot, radar plot and 2D scatter plot with t-Distributed Stochastic Neighbor Embedding	NA
6	2019	Driscoll et al. (2019)	CLARITY-cleared mouse brain neurons (region not specified)	LSM		Machine-learning based supervised spines detection	n/d	u-shape3D software (+)	Unsupervised hierarchical clustering (=9), data dimensionality deduction by 2D principal component analysis (PCA)	NA

*Software availability—NA, not available; UR, available upon request; FA, freely available*.

Another group published an unsupervised construction of the spine shape taxonomy in the same year (Bokota et al., 2016; Table 1). As a first step in the analysis, 11 features that have been most often used in previous publications were extracted from 2D projections of confocal image stacks (Figure 3B). Furthermore, the PCA method was applied to reduce the data dimensionality. Two components were generated, representing a linear combination of six features from 11 initially selected, while five others were neglected due to their relative insignificance. This provides a 2D (or 3D) orthogonal basis, where each spine can be presented as a point at the intersection of corresponding values of two generated components. In this research, dendritic spines in the control group and after chemical LTP induction were compared at two time points with 10 min difference. Only 300 pairs of dendritic spines with the closest meanings of selected parameters were used for further analysis. The authors reasoned that such dataset normalization was due to the initial high diversity of the spine population. Clusterization was performed for spines from both groups at two time points together to build shape taxonomy. Two well-established algorithms, c-means and average-linkage hierarchical representing crisp and fuzzy types of clustering were used. In each case, 11 clusters were formed, but their shape and content were different. Clusters vary greatly from small peripheral to overcrowded, while some clusters were well represented and separated from each other.

To analyze changes in dendritic spine morphology over time, the authors generated a transition shape model, which calculated the probability of dendritic spine transition from one taxonomic unit to another, visually presented as a transition graph. The statistical difference between the resulting models for the control and cLTP groups were analyzed with bootstrap-based statistical tests. The statistics depended only on the changes of distribution, demonstrating the differences between models build with c-means clustering, while models build with hierarchical clustering showed significant difference with statistics comparing the transition of spines between shapes. No statistically relevant differences were found between models when transitions between clusters were compared rather than the whole model, probably because of the little number of analyzed spines. The authors concluded that the clustering algorithm greatly influences the result, and therefore should be selected carefully, considering the properties of the data and experiment design. This study is the only study published so far, where different clustering algorithms were compared using the same dataset. This study was also the first that compared the control and experimental group of dendritic spines in terms of clusters.

More recently, spines from two 40 and 85 year old individuals, layer III pyramidal neurons in the cingulate cortex reconstructed in 3D were subjected to clusterization (Luengo-Sanchez et al., 2018; Table 1). Before clusterization, detached or fragmented spines, due to the diffraction limit of confocal microscopy spines, were repaired by semi-supervised mesh processing algorithms, while spines with extreme features were excluded from the data set. To characterize the spines, the surface was divided into regions according to a multiresolution Reeb graph. In total, seven segments, which are presented as ellipses linked to each other, were generated to mimic the geometry of a spine, and their major geometrical aspects such as length, width, size or curvature were used to generate 36 spines morphology parameters (Figure 3C). In order to create a more precise and sophisticated spine model with an additional 18 features, such as spine growth direction, were also used. To get a more in-depth insight into the nature of generated clusters the most representative feature classification rules, based on the RIPPER algorithm, were generated for each cluster, with each spine was attributed to its most probable cluster. However, the authors established that a single rule cannot be used to characterize all the spines within a cluster, and suggested that each cluster should be defined by one, two, or three observable features.

A cluster consisting of short stubby-like spines was the most homogeneous example, while a cluster with long spines with relatively big heads has the highest variability within a cluster. Stubby-like spines were also clearly separated from other spine classes (Bokota et al., 2016). The authors compared the number of spines in each cluster for apical and basal dendrites, and the dependence of spine shapes on distance from the soma and age of subjects. Statistical analysis showed that cluster distribution significantly differs for all investigated cases. A more precise comparison revealed that only some clusters have valuable differences depending on dendritic compartment, age or combination of both, providing the new information of the relationship between spine shape and function. Basal dendrites displayed smaller stubby-like spines, while apical dendrites have more relatively medium and large spines with a distinct head. Individuals younger than 40 tended to have more small spines, while those over 80 years had more big spines. Since small spines are believed to be a «learning spines», serving as sites where new memories are generated, the authors concluded, that younger subjects have more capabilities of learning, which requires spine plasticity. In addition, the research group evaluated an algorithm generating simulated spines for each cluster using as a basis of 54 morphological features. This simulation reveals the possibility of building a computational model of a pyramidal neuron, which can be used to study neuronal plasticity.

New horizons in the computational analyses of the morphological features of dendritic spines have been created by the application of high-resolution microscopy. Structural illumination microscopy (SIM) enables precise visualization of dendritic spines and measurements of their nanoscale morphological features (Smith et al., 2014). Key morphological descriptors measured on images obtained by SIM are comparable in resolution to electron microscopy. At the same time, SIM provides an ability to analyze many more spines than EM and can also be applied to the analysis of live neurons. SIM microscopy is also able to provide one more meaningful morphological feature, a concave surface: the place where the synaptic junction is thought to be formed. The dynamics of concave surface contacts may define correspondingly dynamic, stable, and degrading synaptic contacts, providing more insight into the potential link between synaptic structure and function.

A method of measuring the nanoscale surface geometry of synaptic spines from SIM images was recently developed (Kashiwagi et al., 2019; Table 1). For the segmentation of spines on SIM images, the combination of Otsu’s multi-level thresholding algorithm with geodesic active contours showed the best result. After that, a polygonal mesh was build basing on spine voxel representation using the marching cube algorithm (Figure 3D). Totally 10 morphological features were extracted from 1,335 dendritic spines, including basic shape features such as length or volume, and more complex parameters obtained by discrete differential-geometry operators such as convex hull volume and open angle. We noticed that the morphological features of large spines could be also measured on neuronal images obtained with high-resolution confocal microscopy with narrow confocal aperture (0.5 AU). Further processing with PCA led to the generation of three components composed of five morphological features (length, volume, convex hull ratio, coefficient of variation in distance, and open angle) covering 93% of data variance.

When analyzing the shapes of spines distributed in 3D feature space, the authors noticed that spines exhibited a continuum of morphologies, supporting the idea that the classification into thin, mushroom, or stubby spines does not reflect the presence of discrete subclasses. The authors also noted that spines with a clearly identifiable head are located close to each other. An SVM classifier with a nonlinear kernel trained on the manually labeled dataset was used to divide spines into mushroom-shaped and non-mushroom. Analysis of spine shapes in a kinase-dead allele of Ca^2+^/calmodulin-dependent protein kinase IIα (CaMKIIαK42R/K42R) knock-in mouse was performed using this approach and it was discovered that the mushroom shaped spines have reduced volume in mutant mice when compared to the control. In contrast, the volume was not changed in non-mushroom spines but their length was significantly elevated in mutant mice samples.

The data obtained during longitudinal SIM imaging of dendritic spine dynamics *in vivo* were also analyzed by the clustering approach. A trajectory was built in 3D feature space, reflecting the changes of spine shape from these data. Interestingly, spines in different parts of feature space showed different patterns of behavior, which enable us to divide them into three groups. The first group predominantly consists of small mushroom spines having short trajectories without an orientation preference, the second group consists of large mushroom spines that moved bidirectionally along the axis, corresponding to medium/thin and large/round shape features, and the third group was composed of non-mushroom spines with highly variable trajectories. The authors concluded that these three groups of spines overlapped in the feature space, and their distribution did not support the existence of distinct shape classes.

In 2019 a new software DXplorer was developed. It enables interactive three-dimensional analysis of dendritic spines morphology (Choi et al., 2019; Table 1). In DXplorer 3D rendering of spines displayed together with the parallel coordinate plot, radar plot, and 2D scatter plot with t-Distributed Stochastic Neighbor Embedding generated in agreement with their high dimensional features. This work is an extension of earlier work (Kashiwagi et al., 2019) and similar methods for the preparation of samples and data acquisition were used in both studies. The authors noticed that using only five morphological features (Kashiwagi et al., 2019) is not enough to distinguish all spines, especially the spines that belong to different types, because spine head and neck dimensions are not included. For example, these features have very close values for some mushroom and stubby spines, and therefore it is impossible to discriminate between them. To overcome this issue five more parameters were added: maximum head diameter (hMax), minimum head diameter (hMin), maximum neck diameter (nMax), minimum neck diameter (nMin), and HNR, which is the ratio of head to the neck (hMax/nMax). To find similar 3D phenotypes, users can group spines with similar shapes through interactive selection using the feature and similarity plot. In addition, the similarity plot panel can be used to divide the spines using the k-means clustering algorithm into a certain number of clusters, which are larger than groups formed in 2D feature space using t-Distributed Stochastic Neighbor Embedding method. Analysis of classification accuracy in 2D by rendering the classified spines in 3D by expert neuroscientists showed that 2D image-based classification had an error rate of 44.27% in identifying spines shapes. The errors occurred most often when stubby spines were labeled as thin spines in 2D projection. This experiment demonstrated that 2D image-based classification has a very high error rate, and therefore analysis in 3D is required to characterize spine shapes correctly.

The application of light sheet microscopy (LSM) enables the collection of morphological information from an intact brain or a large portion of it without physical separation. In a recent application of this technology, a cleared-tissue axially swept light-sheet microscopy (ctASLM; Chakraborty et al., 2019) was used to collect imaging data using mouse brain precleared with PEGASOS method. The dendritic protrusions on these images were analyzed with u-shape3D free available software (Driscoll et al., 2019). As a result, nine dendritic spine clusters with similar shapes were generated after unsupervised hierarchical clustering based on morphological measures, such as the ratio of the spine neck area to the spine surface area. The distribution of obtained classes is shown in the 2D feature space generated by PCA. The development of spine clusterization approaches based on LSM data is a very promising future direction of this research.

## Concluding Remarks

The examples discussed above support the conclusion that the dendritic spine clusterization approach reflects a continuum of spine shapes and sizes much better than classification into predefined groups. The application of the clusterization approach enables more precise analysis and reveals qualitatively new information about synaptic spines. All of the groups discussed above concluded that continuous morphological variables rather than pre-defined spine classes should be used to describe spines morphologies. The clustering approach aims to identify and group objects with similar shapes, with different shape classes determined by the structure of the data. Different clustering algorithms give different results on the same dataset, so clusters vary in shape and content, which may greatly influence the interpretation of these data. Future research will be needed to compare existing methods and identify optimal approaches to clusterization (Table 1). These algorithms should offer maximal discrimination of clusters that facilitate subsequent analysis and identification of the differences between control and experimental groups. It is also possible that a new and superior clusterization algorithm will be developed for this purpose in the future.

As discussed above, there is no consensus on which spine shape descriptors should be used as input for the clusterization procedure. Taking into account the results of the approaches discussed here, we propose that this set of parameters should include not only obvious morphological metrics such as spine head diameter, spine area, and volume but also complex geometrical features that enable a more sophisticated description of complex dendritic spines shapes. Proper statistical procedures for comparing clustered spine data from control and experimental groups need to be developed in the future.

Another important issue is the biological interpretation of clusterization data. In the classification approach putative biological functions of “mushroom,” “thin,” and “stubby” spines have been extensively discussed. Further research will be needed to relate the complex shapes of a particular cluster of spines to its physiological role.

Despite all these challenges, this emerging approach to the analysis of dendritic spine shapes opens a new and powerful trend in neuroscience research. The availability of high quality, free, and robust software is critical for these ideas to become reality. Once developed, such tools will greatly facilitate the investigation of dendritic spines, including function, structure, plasticity, and pathology. Providing datasets, distributives, and source code by developers is an essential step to speed up this process and to open the way to further improvement and adaptation of these methods.

## Author Contributions

The manuscript was drafted and revised for intellectual content by EP and IB, with material support from IB. All authors contributed to the article and approved the submitted version.

## Conflict of Interest

The authors declare that the research was conducted in the absence of any commercial or financial relationships that could be construed as a potential conflict of interest.
